# Methyl 2-{[(2-fur­yl)(3-methyl-5-oxo-1-phenyl-4,5-dihydro-1*H*-pyrazol-4-yl­idene)meth­yl]amino}­acetate

**DOI:** 10.1107/S1600536811026158

**Published:** 2011-07-06

**Authors:** Xiao Han, Xiao-Dong Yang, Xiao-Chang Dai

**Affiliations:** a School of Chemical Science and Technology, Yunnan University, Kunming 65009, People’s Republic of China

## Abstract

In the title compound, C_18_H_17_N_3_O_4_, the amino group of the glycine methyl ester fragment is involved in an intra­molecular N—H⋯O hydrogen bond. The phenyl and furyl rings form dihedral angles of 10.20 (4) and 54.56°, respectively, with the pyrazole ring. In the crystal, mol­ecules related by translation along the *b* axis are linked into chains *via* weak inter­molecular C—H⋯O hydrogen bonds.

## Related literature

For a related structure, see: Zhang *et al.* (2007 ▶). For details of the synthesis, see: Jensen (1959 ▶). For applications of pyrazol­one derivatives in coordination chemistry, see: Casas *et al.* (2007 ▶). For the anti­bacterial activity of pyrazolone derivatives, see: Li *et al.* (2000 ▶); Zhang *et al.* (2008 ▶).
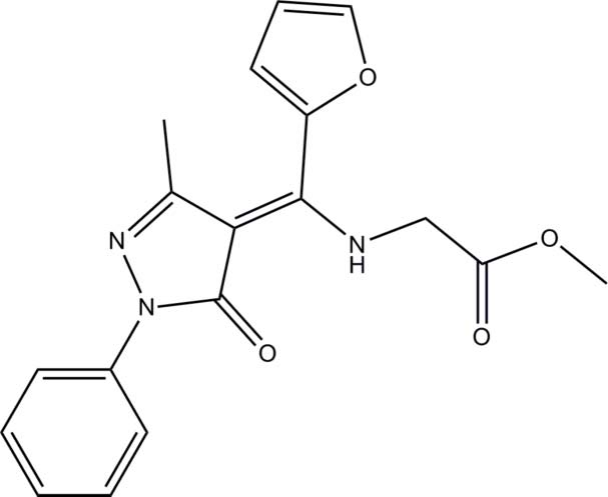


## Experimental

### 

#### Crystal data

C_18_H_17_N_3_O_4_
*M* *_r_* = 339.35
Triclinic, 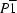

*a* = 7.499 (4) Å
*b* = 9.503 (5) Å
*c* = 11.749 (6) Å
α = 96.712 (7)°
β = 91.654 (8)°
γ = 90.337 (7)°
*V* = 831.2 (7) Å^3^
*Z* = 2
Mo *K*α radiation
μ = 0.10 mm^−1^
*T* = 295 K
0.48 × 0.14 × 0.08 mm

#### Data collection

Bruker APEXII CCD diffractometer
6492 measured reflections
2915 independent reflections
1805 reflections with *I* > 2σ(*I*)
*R*_int_ = 0.034

#### Refinement

*R*[*F* ^2^ > 2σ(*F* ^2^)] = 0.051
*wR*(*F* ^2^) = 0.150
*S* = 1.01
29015 reflections
232 parameters
H atoms treated by a mixture of independent and constrained refinement
Δρ_max_ = 0.17 e Å^−3^
Δρ_min_ = −0.21 e Å^−3^

### 

Data collection: *APEX2* (Bruker, 2005 ▶); cell refinement: *SAINT-Plus* (Bruker, 2005 ▶); data reduction: *SAINT-Plus*; program(s) used to solve structure: *SHELXTL* (Sheldrick, 2008 ▶); program(s) used to refine structure: *SHELXTL*; molecular graphics: *SHELXTL*; software used to prepare material for publication: *SHELXTL*.

## Supplementary Material

Crystal structure: contains datablock(s) I, global. DOI: 10.1107/S1600536811026158/cv5123sup1.cif

Structure factors: contains datablock(s) I. DOI: 10.1107/S1600536811026158/cv5123Isup2.hkl

Supplementary material file. DOI: 10.1107/S1600536811026158/cv5123Isup3.cml

Additional supplementary materials:  crystallographic information; 3D view; checkCIF report

## Figures and Tables

**Table 1:** Hydrogen-bond geometry (Å, °)

*D*—H⋯*A*	*D*—H	H⋯*A*	*D*⋯*A*	*D*—H⋯*A*
N3—H3*A*⋯O1	0.97 (3)	1.89 (3)	2.704 (3)	140 (2)
C14—H14⋯O1^i^	0.93	2.48	3.386 (4)	164

Symmetry code: (i) 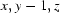.

**Fig. 1.**
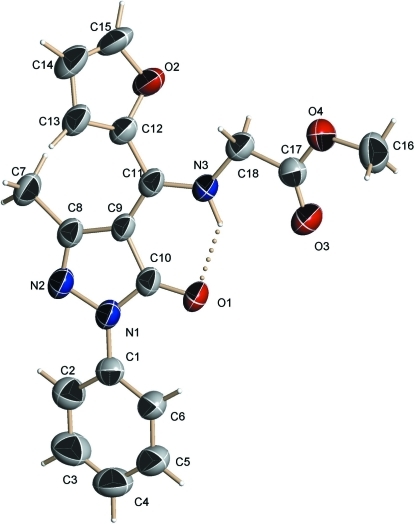
The molecular structure of (I) showing the atomic numbering and 30% probability displacement ellipsoids. Dotted line indicates hydrogen bond.

## supplementary crystallographic information

### Comment

Pyrazolones constitute an important class of heterocycles due to their properties and applications (Casas *et al.*, 2007). Schiff bases derived from 1-phenyl-3-methyl-4-(2-furoyl)-5-pyrazolone (PMFP) have found extensive application in coordination chemistry and due to their antibacterial activity (Zhang *et al.*, 2007, 2008; Li *et al.*, 2000). In order to expand this field, we present here the title compound (I).

In (I) (Fig. 1), the phenyl ring (C1-C6) is twisted at 10.20 (4)° from the mean plane of pyrazole ring. The pyrazole ring and the O1/C10/C9/C11/N3 mean form a dihedral angle of 5.62 (4)°. The bond length of C9—C11(1.390 (3) Å) between the usual C—C and C=C bonds indicates the delocalization of the electrons. Strong intramolecular hydrogen bond N3—H3A···O1(Table 1) is indicative of the enamine-keto form.

In the crystal structure, molecules related by translation along axis *b* are linked into chains *via* weak intermolecular C—H···O hydrogen bonds.

### Experimental

PMFP was synthesized according to the method proposed by Jensen (1959). A mixture of a 10 ml PMFP (2 mmol, 0.5366 g) anhydrous ethanol solution, and 10 ml Glycine methyl ester hydrochloride anhydrous ethanol solution (2 mmol, 0.2511 g)solution was refluxed for *ca*.7 h, adding a few drops of glacial acetic acid as a catalyst. Then ethanol was removed by evaporation and the resulting black precipitate formed was filtered off, washed with cold anhydrous ethanol and dried in air. Black block single crystals suitable for analysis were obtained by slowly evaporation of a solution in anhydrous ethanol at room temperature for a few days.

### Refinement

H atoms bonded to N3 was located in a difference map and isotropically refined. Other H atoms were placed in calculated positions [C—H 0.93-0.97 Å], and refined as riding, with U_iso_(H)=1.2-1.5 U_eq_(C).

### Crystal data

**Table:**

C_18_H_17_N_3_O_4_	*Z* = 2
*M**_r_* = 339.35	*F*(000) = 356
Triclinic, *P*1	*D*_x_ = 1.356 Mg m^−^^3^
Hall symbol: -P 1	Mo *K*α radiation, λ = 0.71073 Å
*a* = 7.499 (4) Å	Cell parameters from 1502 reflections
*b* = 9.503 (5) Å	θ = 2.9–22.6°
*c* = 11.749 (6) Å	µ = 0.10 mm^−^^1^
α = 96.712 (7)°	*T* = 295 K
β = 91.654 (8)°	Block, black
γ = 90.337 (7)°	0.48 × 0.14 × 0.08 mm
*V* = 831.2 (7) Å^3^	

### Data collection

**Table:**

Bruker APEXII CCD diffractometer	1805 reflections with *I* > 2σ(*I*)
Radiation source: fine-focus sealed tube	*R*_int_ = 0.034
graphite	θ_max_ = 25.0°, θ_min_ = 2.6°
phi and ω scans	*h* = −8→8
6492 measured reflections	*k* = −11→11
2915 independent reflections	*l* = −13→13

### Refinement

**Table:**

Refinement on *F*^2^	Primary atom site location: structure-invariant direct methods
Least-squares matrix: full	Secondary atom site location: difference Fourier map
*R*[*F*^2^ > 2σ(*F*^2^)] = 0.051	Hydrogen site location: inferred from neighbouring sites
*wR*(*F*^2^) = 0.150	H atoms treated by a mixture of independent and constrained refinement
*S* = 1.01	*w* = 1/[σ^2^(*F*_o_^2^) + (0.0826*P*)^2^] where *P* = (*F*_o_^2^ + 2*F*_c_^2^)/3
2915 reflections	(Δ/σ)_max_ < 0.001
232 parameters	Δρ_max_ = 0.17 e Å^−^^3^
0 restraints	Δρ_min_ = −0.21 e Å^−^^3^

### Special details

**Table:**

Geometry. All e.s.d.'s (except the e.s.d. in the dihedral angle between two l.s. planes) are estimated using the full covariance matrix. The cell e.s.d.'s are taken into account individually in the estimation of e.s.d.'s in distances, angles and torsion angles; correlations between e.s.d.'s in cell parameters are only used when they are defined by crystal symmetry. An approximate (isotropic) treatment of cell e.s.d.'s is used for estimating e.s.d.'s involving l.s. planes.
Refinement. Refinement of *F*^2^ against ALL reflections. The weighted *R*-factor *wR* and goodness of fit *S* are based on *F*^2^, conventional *R*-factors *R* are based on *F*, with *F* set to zero for negative *F*^2^. The threshold expression of *F*^2^ > σ(*F*^2^) is used only for calculating *R*-factors(gt) *etc*. and is not relevant to the choice of reflections for refinement. *R*-factors based on *F*^2^ are statistically about twice as large as those based on *F*, and *R*- factors based on ALL data will be even larger.

### Fractional atomic coordinates and isotropic or equivalent isotropic displacement parameters (Å^2^)

**Table:**

	*x*	*y*	*z*	*U*_iso_*/*U*_eq_	
C1	0.2043 (3)	0.1986 (3)	0.7105 (2)	0.0509 (6)	
C2	0.1118 (4)	0.1820 (3)	0.6063 (3)	0.0697 (8)	
H2	0.0454	0.1000	0.5838	0.084*	
C3	0.1192 (5)	0.2881 (4)	0.5362 (3)	0.0888 (10)	
H3	0.0555	0.2778	0.4666	0.107*	
C4	0.2192 (5)	0.4093 (4)	0.5672 (3)	0.0848 (10)	
H4	0.2235	0.4799	0.5188	0.102*	
C5	0.3120 (4)	0.4247 (3)	0.6696 (3)	0.0725 (8)	
H5	0.3797	0.5064	0.6909	0.087*	
C6	0.3064 (4)	0.3203 (3)	0.7418 (2)	0.0599 (7)	
H6	0.3707	0.3313	0.8112	0.072*	
C7	0.0294 (4)	−0.2611 (3)	0.8061 (2)	0.0648 (8)	
H7A	−0.0553	−0.2639	0.7430	0.097*	
H7B	−0.0311	−0.2784	0.8741	0.097*	
H7C	0.1178	−0.3325	0.7892	0.097*	
C8	0.1184 (3)	−0.1175 (2)	0.8250 (2)	0.0493 (6)	
C9	0.2063 (3)	−0.0467 (2)	0.9256 (2)	0.0446 (6)	
C10	0.2473 (3)	0.0934 (2)	0.8965 (2)	0.0464 (6)	
C11	0.2438 (3)	−0.0855 (2)	1.0341 (2)	0.0461 (6)	
C12	0.2315 (3)	−0.2330 (2)	1.0582 (2)	0.0507 (6)	
C13	0.2946 (3)	−0.3534 (2)	1.0035 (2)	0.0605 (7)	
H13	0.3546	−0.3628	0.9349	0.073*	
C14	0.2530 (4)	−0.4631 (3)	1.0695 (3)	0.0741 (9)	
H14	0.2804	−0.5585	1.0534	0.089*	
C15	0.1673 (4)	−0.4029 (3)	1.1587 (3)	0.0827 (10)	
H15	0.1234	−0.4516	1.2163	0.099*	
C16	0.6100 (4)	0.1991 (3)	1.4644 (3)	0.0859 (10)	
H16A	0.7236	0.2106	1.4309	0.129*	
H16B	0.6277	0.1728	1.5403	0.129*	
H16C	0.5461	0.2867	1.4681	0.129*	
C17	0.4484 (3)	0.1167 (3)	1.2930 (2)	0.0533 (6)	
C18	0.3474 (3)	−0.0089 (2)	1.2339 (2)	0.0538 (7)	
H18A	0.4205	−0.0928	1.2334	0.065*	
H18B	0.2405	−0.0242	1.2756	0.065*	
H3A	0.309 (3)	0.107 (3)	1.094 (2)	0.065 (8)*	
N1	0.1955 (2)	0.09022 (19)	0.78289 (18)	0.0501 (5)	
N2	0.1101 (3)	−0.0388 (2)	0.74126 (18)	0.0540 (6)	
N3	0.2991 (3)	0.0135 (2)	1.11800 (17)	0.0498 (5)	
O1	0.3157 (2)	0.19626 (16)	0.95855 (14)	0.0587 (5)	
O2	0.1511 (3)	−0.26029 (18)	1.15579 (17)	0.0717 (6)	
O3	0.4695 (3)	0.22735 (19)	1.25441 (17)	0.0740 (6)	
O4	0.5077 (2)	0.08888 (19)	1.39454 (17)	0.0684 (5)	

### Atomic displacement parameters (Å^2^)

**Table:**

	*U*^11^	*U*^22^	*U*^33^	*U*^12^	*U*^13^	*U*^23^
C1	0.0540 (14)	0.0451 (14)	0.0541 (16)	0.0075 (11)	0.0107 (12)	0.0048 (12)
C2	0.0727 (18)	0.0695 (19)	0.067 (2)	−0.0029 (15)	−0.0007 (16)	0.0103 (16)
C3	0.099 (2)	0.098 (3)	0.073 (2)	0.001 (2)	−0.0079 (19)	0.029 (2)
C4	0.103 (2)	0.071 (2)	0.086 (3)	0.0040 (19)	0.010 (2)	0.0325 (19)
C5	0.090 (2)	0.0497 (17)	0.079 (2)	0.0030 (15)	0.0129 (18)	0.0110 (15)
C6	0.0764 (18)	0.0431 (15)	0.0602 (17)	0.0008 (13)	0.0075 (14)	0.0051 (13)
C7	0.0724 (17)	0.0433 (15)	0.0753 (19)	−0.0130 (13)	0.0006 (15)	−0.0057 (13)
C8	0.0486 (14)	0.0396 (13)	0.0582 (16)	0.0015 (11)	0.0102 (12)	−0.0031 (13)
C9	0.0488 (13)	0.0317 (12)	0.0516 (15)	0.0004 (10)	0.0073 (11)	−0.0037 (11)
C10	0.0476 (13)	0.0376 (13)	0.0523 (16)	0.0022 (10)	0.0064 (11)	−0.0037 (11)
C11	0.0430 (13)	0.0352 (13)	0.0589 (16)	0.0015 (10)	0.0111 (11)	−0.0029 (12)
C12	0.0510 (14)	0.0375 (13)	0.0633 (16)	−0.0027 (11)	0.0126 (12)	0.0017 (11)
C13	0.0667 (16)	0.0362 (14)	0.0769 (18)	0.0007 (12)	0.0145 (14)	−0.0040 (13)
C14	0.080 (2)	0.0348 (15)	0.107 (2)	0.0035 (13)	0.0123 (18)	0.0032 (15)
C15	0.107 (2)	0.0366 (16)	0.107 (3)	−0.0150 (15)	0.020 (2)	0.0167 (16)
C16	0.093 (2)	0.090 (2)	0.068 (2)	−0.0136 (19)	−0.0048 (17)	−0.0150 (18)
C17	0.0572 (15)	0.0503 (16)	0.0520 (17)	0.0034 (12)	0.0090 (13)	0.0026 (13)
C18	0.0562 (15)	0.0431 (14)	0.0618 (18)	−0.0002 (11)	0.0050 (13)	0.0046 (12)
N1	0.0560 (12)	0.0388 (11)	0.0533 (13)	−0.0014 (9)	0.0035 (10)	−0.0036 (10)
N2	0.0573 (13)	0.0410 (12)	0.0612 (14)	−0.0026 (9)	0.0054 (10)	−0.0052 (11)
N3	0.0633 (13)	0.0348 (11)	0.0507 (13)	0.0006 (9)	0.0020 (10)	0.0025 (10)
O1	0.0786 (12)	0.0353 (9)	0.0598 (11)	−0.0064 (8)	−0.0016 (9)	−0.0035 (8)
O2	0.0906 (13)	0.0409 (10)	0.0846 (14)	−0.0075 (9)	0.0310 (11)	0.0049 (9)
O3	0.0962 (14)	0.0482 (12)	0.0768 (14)	−0.0107 (10)	−0.0018 (11)	0.0063 (10)
O4	0.0828 (13)	0.0657 (13)	0.0548 (12)	−0.0119 (10)	−0.0029 (10)	0.0016 (10)

### Geometric parameters (Å, °)

**Table:**

C1—C2	1.382 (4)	C11—N3	1.335 (3)
C1—C6	1.390 (4)	C11—C12	1.464 (3)
C1—N1	1.412 (3)	C12—C13	1.341 (3)
C2—C3	1.377 (4)	C12—O2	1.361 (3)
C2—H2	0.9300	C13—C14	1.408 (4)
C3—C4	1.377 (5)	C13—H13	0.9300
C3—H3	0.9300	C14—C15	1.320 (4)
C4—C5	1.365 (4)	C14—H14	0.9300
C4—H4	0.9300	C15—O2	1.366 (3)
C5—C6	1.380 (4)	C15—H15	0.9300
C5—H5	0.9300	C16—O4	1.453 (3)
C6—H6	0.9300	C16—H16A	0.9600
C7—C8	1.505 (3)	C16—H16B	0.9600
C7—H7A	0.9600	C16—H16C	0.9600
C7—H7B	0.9600	C17—O3	1.204 (3)
C7—H7C	0.9600	C17—O4	1.317 (3)
C8—N2	1.303 (3)	C17—C18	1.498 (4)
C8—C9	1.431 (3)	C18—N3	1.440 (3)
C9—C11	1.390 (3)	C18—H18A	0.9700
C9—C10	1.446 (3)	C18—H18B	0.9700
C10—O1	1.247 (3)	N1—N2	1.409 (3)
C10—N1	1.377 (3)	N3—H3A	0.97 (3)
			
C2—C1—C6	119.7 (2)	C13—C12—O2	109.9 (2)
C2—C1—N1	119.3 (2)	C13—C12—C11	131.8 (2)
C6—C1—N1	121.1 (2)	O2—C12—C11	118.2 (2)
C3—C2—C1	119.3 (3)	C12—C13—C14	107.2 (2)
C3—C2—H2	120.4	C12—C13—H13	126.4
C1—C2—H2	120.4	C14—C13—H13	126.4
C2—C3—C4	121.2 (3)	C15—C14—C13	106.1 (2)
C2—C3—H3	119.4	C15—C14—H14	127.0
C4—C3—H3	119.4	C13—C14—H14	127.0
C5—C4—C3	119.4 (3)	C14—C15—O2	111.5 (3)
C5—C4—H4	120.3	C14—C15—H15	124.2
C3—C4—H4	120.3	O2—C15—H15	124.2
C4—C5—C6	120.6 (3)	O4—C16—H16A	109.5
C4—C5—H5	119.7	O4—C16—H16B	109.5
C6—C5—H5	119.7	H16A—C16—H16B	109.5
C5—C6—C1	119.8 (3)	O4—C16—H16C	109.5
C5—C6—H6	120.1	H16A—C16—H16C	109.5
C1—C6—H6	120.1	H16B—C16—H16C	109.5
C8—C7—H7A	109.5	O3—C17—O4	125.1 (3)
C8—C7—H7B	109.5	O3—C17—C18	125.1 (3)
H7A—C7—H7B	109.5	O4—C17—C18	109.8 (2)
C8—C7—H7C	109.5	N3—C18—C17	110.5 (2)
H7A—C7—H7C	109.5	N3—C18—H18A	109.6
H7B—C7—H7C	109.5	C17—C18—H18A	109.6
N2—C8—C9	112.3 (2)	N3—C18—H18B	109.6
N2—C8—C7	117.8 (2)	C17—C18—H18B	109.6
C9—C8—C7	129.8 (2)	H18A—C18—H18B	108.1
C11—C9—C8	133.2 (2)	C10—N1—N2	111.56 (19)
C11—C9—C10	121.9 (2)	C10—N1—C1	129.3 (2)
C8—C9—C10	104.8 (2)	N2—N1—C1	118.9 (2)
O1—C10—N1	126.3 (2)	C8—N2—N1	106.2 (2)
O1—C10—C9	128.8 (2)	C11—N3—C18	126.3 (2)
N1—C10—C9	104.9 (2)	C11—N3—H3A	113.8 (15)
N3—C11—C9	119.2 (2)	C18—N3—H3A	119.8 (15)
N3—C11—C12	118.9 (2)	C12—O2—C15	105.4 (2)
C9—C11—C12	121.9 (2)	C17—O4—C16	117.5 (2)
			
C6—C1—C2—C3	−1.5 (4)	C11—C12—C13—C14	176.1 (3)
N1—C1—C2—C3	179.4 (2)	C12—C13—C14—C15	0.4 (3)
C1—C2—C3—C4	1.1 (5)	C13—C14—C15—O2	−0.4 (4)
C2—C3—C4—C5	−0.4 (5)	O3—C17—C18—N3	7.8 (4)
C3—C4—C5—C6	0.0 (5)	O4—C17—C18—N3	−173.49 (19)
C4—C5—C6—C1	−0.5 (4)	O1—C10—N1—N2	−175.3 (2)
C2—C1—C6—C5	1.2 (4)	C9—C10—N1—N2	5.2 (2)
N1—C1—C6—C5	−179.7 (2)	O1—C10—N1—C1	−0.7 (4)
N2—C8—C9—C11	178.9 (2)	C9—C10—N1—C1	179.8 (2)
C7—C8—C9—C11	2.6 (4)	C2—C1—N1—C10	−166.8 (2)
N2—C8—C9—C10	2.5 (3)	C6—C1—N1—C10	14.1 (4)
C7—C8—C9—C10	−173.8 (2)	C2—C1—N1—N2	7.4 (3)
C11—C9—C10—O1	−0.9 (4)	C6—C1—N1—N2	−171.6 (2)
C8—C9—C10—O1	175.9 (2)	C9—C8—N2—N1	0.6 (2)
C11—C9—C10—N1	178.59 (19)	C7—C8—N2—N1	177.36 (19)
C8—C9—C10—N1	−4.5 (2)	C10—N1—N2—C8	−3.7 (2)
C8—C9—C11—N3	−167.5 (2)	C1—N1—N2—C8	−178.93 (18)
C10—C9—C11—N3	8.3 (3)	C9—C11—N3—C18	−178.5 (2)
C8—C9—C11—C12	15.0 (4)	C12—C11—N3—C18	−0.9 (3)
C10—C9—C11—C12	−169.2 (2)	C17—C18—N3—C11	164.5 (2)
N3—C11—C12—C13	−129.3 (3)	C13—C12—O2—C15	−0.1 (3)
C9—C11—C12—C13	48.2 (4)	C11—C12—O2—C15	−176.9 (2)
N3—C11—C12—O2	46.7 (3)	C14—C15—O2—C12	0.3 (3)
C9—C11—C12—O2	−135.8 (2)	O3—C17—O4—C16	−1.5 (4)
O2—C12—C13—C14	−0.2 (3)	C18—C17—O4—C16	179.8 (2)

### Hydrogen-bond geometry (Å, °)

**Table:**

*D*—H···*A*	*D*—H	H···*A*	*D*···*A*	*D*—H···*A*
N3—H3A···O1	0.97 (3)	1.89 (3)	2.704 (3)	140 (2)
C14—H14···O1^i^	0.93	2.48	3.386 (4)	164

Symmetry codes: (i) *x*, *y*−1, *z*.
